# Preliminary assessment of the NACA0021 trailing edge wedge for wind turbine application^☆^

**DOI:** 10.1016/j.heliyon.2023.e21193

**Published:** 2023-10-19

**Authors:** Asmail A.M. Abdalkarem, Roaa Ansaf, Wan Khairul Muzammil, Adnan Ibrahim, Zambri Harun, Ahmad Fazlizan

**Affiliations:** a Solar Energy Research Institute (SERI), University Kebangsaan Malaysia, 43600 Bangi, Selangor, Malaysia; b Centre of Research in Energy and Advanced Materials, Faculty of Engineering, University Malaysia Sabah, Jln. UMS, 88400, Kota Kinabalu, Malaysia; c Department of Mechanical and Manufacturing Engineering, Faculty of Engineering and Built Environment, University Kebangsaan Malaysia (UKM), Bangi, 43600, Malaysia

**Keywords:** CFD, Power augmentation, Airfoil, Wedge flap, Wedge tail

## Abstract

The airfoil blade is the primary component of a wind turbine, and its aerodynamic properties play a crucial role in determining the energy conversion efficiency of these blades. Many researchers have proposed different airfoil modifications intending to enhance the aerodynamic characteristics and limit the unsteady interaction with the atmospheric boundary layer. This study evaluates the benefits of mounting wedge tails (WTs) on the trailing edge of an airfoil. The aerodynamic characteristics of a 2-D, steady-state NACA 0021 airfoil featuring the wedge tails (WT) and fish wedge tails (FWT) were studied computationally by employing the shear stress transport (SST) k-ω turbulence model. Different WT and FWT configurations were studied at various wedge length (L) to wedge height (H) ratios, L/H, at the airfoil's trailing edge. The effects of different L/H ratios, including L/H > 1, L/H = 1, and L/H < 1, were considered in the present study to determine the optimal configuration to achieve the maximum glide ratio, C_L_/C_D_ at the Reynolds number of 180,000. The findings indicate that the performance of the NACA 0021 airfoil was notably affected by the height of the tail; however, the length had only a minor impact when L/H was less than 1. The mounted FWT resulted in significant enhancements to both the lift and glide ratio of the airfoil. Specifically, the lift ratio experienced an increase of over 41 % compared to the clean airfoil, while the glide ratio increased by more than 31 %. These improvements were observed at an ideal height and length of 2.5 % and 1 % of the airfoil, respectively. Moreover, the mounted FWT performed better than the Gurney flap using the same configurations.

## 1 Introduction

The horizontal axis wind turbine (HAWT) has received enormous interest from both researchers and industry players worldwide to overcome its many deficiencies. The technology has been developed, and its efficiency has improved, with a new performance rating having been achieved that is close to the Betz limit. In contrast, vertical axis wind turbines (VAWTs) have not received such enormous investment as HAWTs, which has prevented them from competing commercially. Despite the numerous advantages that VAWTs have in comparison to HAWTs, VAWTs nevertheless encounter a range of problems, i.e. [1,2],.

• VAWT blades face a wide range of angles of attack (AOA), periodically at different azimuth angles. This generates a phenomenon called a dynamic stall at the tip speed ratio, TSR <4. This significantly impacts the noise, vibration, and power output of VAWTs.
• The vortices produced by the turbine components interact with each other and with the blades during rotation, causing the highly complicated flow characteristic.

Because of the VAWT design, there is interaction between the blade and the shed vorticity that forms at the upwind route during the blade's downwind passage. The performance of VAWT is adversely affected by these interactions [3]. A complete stall of the VAWT blades and reattachment of the flow are the results of the dynamic stall phenomena in VAWTs, which goes through several stages, i. e, attached flow, creation of the leading edge (LE) vortex, and shedding of the LE vortex [2].

Laneville and Vittecoq [4] experimented on the Darrieus turbine rotating at different TSRs. The results revealed that the dynamic stall occurred at TSR <4, and it was observed that the vortex formed at the LE moved over the airfoil and shed at the trailing edge (TE); it then interacted with the airfoil surface downstream. Sane [5], who is concerned with the aerodynamics of insects' flight and flapping wing motions, concluded that at high AOAs, a prominent LE vortex remains constant in size and attached to the insect wings, and it does not shed into an unsteady wake, as had been expected from non-flapping wings. This occurs through the suppression of the new vorticity generated at the LE, which leads to the generated additional vorticity at the TE stopping, and the airfoil obeys the Kutta condition. The results indicated the need to design a flapping mechanism to suppress the vortices shedding from the LE and TE of the airfoil or wing. Simão Ferreira et al. [6] investigated the creation of dynamic stall of a VAWT at various TSRs using 2-D particle image velocimetry. The findings of the study demonstrate that the flow's growth is contingent upon the size of the AOA and the manner in which the shed vorticity is conveyed and continues to interact with the airfoil or blades. Rezaeiha et al. [7] systematically investigated various impacts of operational parameters on the performance of VAWTs. Their study revealed the inherent unsteadiness of VAWTs, which was attributed to the variations of AOA and relative velocity during each revolution. They also concluded that the main contributor to the total power output is the upwind quartile, using such power improvement methods. The findings suggested that an ideal variable speed operation, such as that employed in HAWTs, may be utilized to regulate the design of VAWTs.

Previous studies indicate that VAWTs can operate at power outputs comparable to HAWTs while the unsteady interactions with the atmospheric boundary layer are reduced. This erratic occurrence, which results in varying torque, is a major reason for poor turbine performance [8]. This nonlinear aspect exists widely in various current applications in aeronautics, hydrodynamics, and wind engineering, mainly on wings and rotating blades. It is sensitive to multiple parameters, such as the Reynolds number (Re), airfoil configuration, solidity parameters, and TSR [9,10]. Hence, the main task is to optimize the VAWT's design to improve its efficiency and maximize the power output [11].

### 1.1 Passive flow control for performance enhancements

Many effective methods were proposed to enhance the performance of turbomachinery, including the VAWT by mitigating dynamic stall. These techniques can be divided based on energy expenditure into passive and active flow control techniques. Several active flow control devices, such as the leading-edge rod, leading-edge slot blowing [12], the blowing jet [13], the oscillating flap [14], the synthetic jet [15,16], and dielectric barrier discharge plasma actuators [17] have been developed and investigated. In general, dynamic stalls can be suppressed using active flow control techniques by modifying the control settings. However, these techniques require complex control mechanisms, maintenance, algorithms, weight, and extra energy expenditure during operations for flow structure improvements, which need to be outweighed by any benefits obtained. In contrast, passive flow control techniques, cited by, for example [18,19], include the LE micro cylinder [,[20], [21], Gurney flaps (GFs) [22], a thin plate at the TE [23], LE serrations [24], an airfoil with a cavity [25], the variable droop LE [26], the LE slat [27], and vortex generators [28,29]. Moreover, controlling the flow using riblet surfaces [30,31] is usually cheaper and easier to install, and no further energy is needed.

The Gurney flap is characterized by its simplicity and ease of fabrication. In several works in the literature on wind turbines, GFs have been proposed as a promising flow control and performance-enhancement technology. They have been used in various industrial applications such as airfoils, wings, aircraft, helicopter rotors, and wind turbines. Many researchers have provided overviews of GF applications, such as Wang et al. [32]. The summary of previous research on the effect of a GF to straight-blade VAWT performance is as follows.

• Zhu et al. [33] studied the effects of two passive control devices, GF with and without dimple, on the performance of straight-bladed VAWT with a NACA 0021 airfoil by using various inboard and outboard configurations at a fixed GF height of 1.25%c. They concluded that the outboard GF and outboard dimple GF obtained better power coefficients.
• Bianchini et al. [34] evaluated numerically the use of NACA 0021 with GF for power enhancement of a VAWT at different TSRs and GF heights. The tangential force was shown to be efficiently increased by the GF in the upstream region and decreased in the downstream region.
• Yan et al. [35] performed numerical simulations of the 2D airfoil NACA 0018 and an SB-VAWT to investigate the effect of the inboard GF with heights of 1%c to 5%c. Their results showed that the optimal GF decreased with varying azimuth angles from upstream to downstream.
• Ni et al. [35] investigated numerically the effects of GFs on the aerodynamic performance of SB-VAWTs with an NACA 0021. The researchers came to the conclusion that, in comparison to the clean airfoil, adding an outboard GF with 2%c on the outside of the blade could improve performance during the upwind half of the blade's rotation. Downwind, however, half the drag force increased, resulting in similar or slightly worse performance during the entire revolution.
• Wiśniewski et al. [36] evaluated the effects of adding GFs up to 5%c as a function of different rotor solidity levels, the NACA 00xx series, and the geometry of the GF itself inward, outward, and fishtail (FT) (both sides of the GF at 45°) on the performance of static airfoil conditions and in a dynamic VAWT. Their results revealed that the outward orientation of the GF provided the most outstanding turbine efficiency at a fixed TSR; however, in the case of the outward position, the torque distribution led to a more significant imbalance between the upwind and downwind halves of the turbine, while the FT configurations provided highly balanced torque between the downwind and upwind halves of the wind turbine. However, the power coefficient was shown to increase less at the upwind half.
• Balduzzi et al. [37] studied experimentally and numerically how aerodynamic behavior was impacted by three different geometrical GF configurations (1.4%c, 2.5%c, and with an inclination angle of 45^օ^ at both sides), added to the NACA 0021 airfoil under both static and dynamic conditions, at AOAs ranging from 0^օ^ to 180^օ^ and Re = 180k. The results concluded that the GF with the 2.5%c height configuration provided the maximum increase of both the C_L_ and C_L_/C_D_ ratio, while the FT2.5 % provided the maximum rise of C_L_ for both positive and negative incidence.
• Mousavi et al. [38] investigated computationally the effects of the GF angle and various GF configurations, including one-sided and two-sided GFs, on the performance of a VAWT. Their results indicated that the inclined two-sided GF performed better at low TSRs but not at a high range of TSRs. The authors recommended adding a controllable GF, which could be deployed at certain TSRs.
• Zhu et al. [39] investigated numerically how the geometric parameters (height and width) of an outboard GF ranging from 0.5%c to 1.75%c affected the performance of an SB-VAWT with a NACA 0018. They found that a short GF blade was more appropriate than a high GF, and it could effectively limit the deficiency of the aerodynamic loss in the downstream half.

From the above literature, it can be concluded that the optimal GF decreased with TSR increases and varying azimuth angles from upstream to downstream, resulting in the GF having a negative impact in the downstream half. Meanwhile, the fishtail configuration showed very balanced positive torque between the wind turbine's downwind and upwind halves. These findings are significant for overall efficiency, as well as the possible creation of stresses and vibrations due to unsteadiness.

### 1.2 Purpose of the current work

The previous literature shows different attempts and recommendations to reduce dynamic stall and wake interactions, as well as their impacts on the downwind half of VAWTs. The work by Wiśniewski [36] showed stunning results in terms of positive, balanced torque. However, the power was reduced at the upwind half of the VAWT compared to one side of the GF.

This study provides the initial evaluation of the impact of utilizing a 2D static NACA 0021 in conjunction with a two-sided wedge flap mounted at a 90° angle, named a wedge tail (WT), and a two-sided wedge flap with an inclination angle of 45°, called a fish wedge tail (FWT). The airfoil modification aimed to overcome the power degradation that occurs when it is applied to the VAWT. The effects of modified airfoils on aerodynamic performance were investigated computationally using ANSYS FLUENT software at various angle of attacks (AOAs). The analysis involved determining the optimal dimensions (height and length) for both the WT and FWT to achieve maximum aerodynamic performance. This information can be adapted to the VAWT design to incorporate the optimal wedge tail size.

## 2 Computational simulation methodology

Airfoil blades, the main elements of wind turbines, interact with wind and convert the kinetic energy from the air to force components, which create the rotation and produce mechanical energy on the shaft. The higher the airfoil's glide ratio (lift coefficient to drag coefficient), the greater the wind energy conversion efficiency. Thus, the glide ratio is the benchmark that determines the energy conversion efficiency of wind turbines [40].

**Fig. 1:**
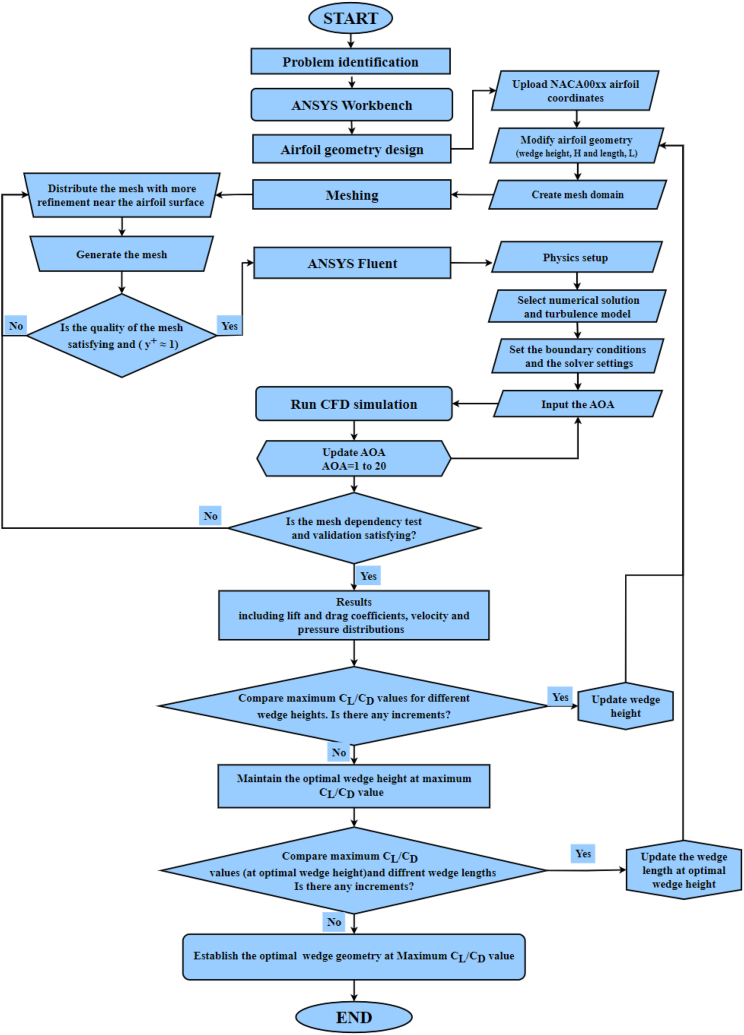
CFD simulation procedure.

In this study, the ANSYS Fluent software is utilized as a solver platform for computational simulations. The solver solves the governing mathematical equations to anticipate fluid flow phenomena using the finite volume method. An overview of the sequence steps followed in CFD Modeling is shown in Fig. 1.

### 2.1 Airfoil profile modifications

The symmetric four-digit NACA airfoil series finds extensive use in VAWT applications and is recognized for its relatively thick profile, which enhances wind turbine performance [41]. The performance of an SB-VAWT using symmetric and non-symmetric NACA airfoils was studied numerically using CFD simulation. It was observed that the NACA 0021 performed best at TSRs <4 among the symmetric NACA00xx series studied. Du et al. [42] tested experimentally three‐bladed H‐Darrieus turbines with various airfoil profiles. They concluded that the NACA 0021 profile contributes to better self-starting capability, thus enhancing the power coefficient at low TSRs. The current study chose the NACA 0021 airfoil with a chord length (c) of 140 mm. This particular airfoil has undergone both experimental and computational examinations by several researchers in the past [37,43,44]. The effects of various WT and FWT dimensions were simulated and benchmarked against the clean airfoil case as follows:.

**Fig. 2:**
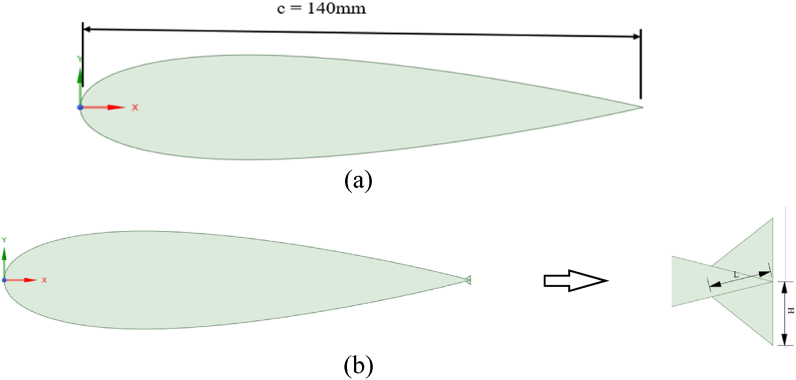
Cross-section the NACA 0021 (a) Clean airfoil; (b) Airfoil with TE-WT.

**Fig. 3:**
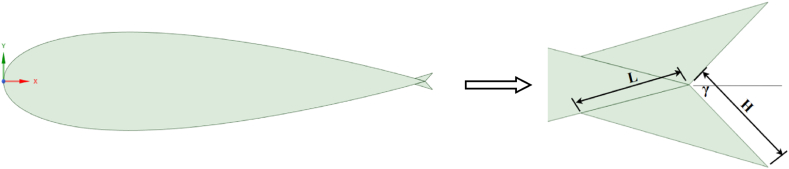
Airfoil geometry of NACA 0021 with TE-FWT.

**Fig. 4:**
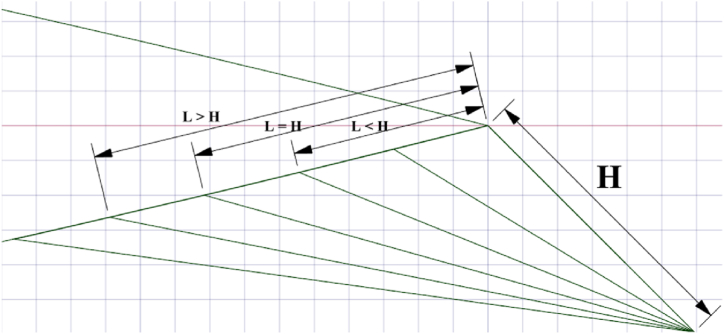
Schematic view of wedge flap with different L/H ratios.

• Effects of the TE-WT height: Fig. 2 shows the geometric difference between clean NACA 0021 airfoil (Fig. 2(a)) and the modified NACA 0021 airfoil with the WT (Fig. 2(b)), where L refers to the length of the WT extended upstream, and represents the vertical distance from the midpoint of the WT to the chord line. Various WT heights were investigated to identify the optimal height corresponding to the maximum (C_L_/C_D_) values. The dimensions of the WFs were defined relative to the airfoil chord. In this section, the inward and outward halves of the WFs were symmetrical and had the same dimensions, H = L.

• Effects of the TE-FWT height: The modified NACA 0021 airfoil with the FWT is shown in Fig. 3. Both wedges inward and outward of the FWT were symmetrical and had the same dimensions, H = L, where γ is the mounting angle (45^օ^) between the WF and the airfoil chord line. Various FWT heights were examined, with the optimal WT height being considered at the highest (C_L_/C_D_) values.

• Effects of the WT lengths: The impact of varying lengths on the optimal constant wedge height, as obtained in the preceding section, is studied in determining the optimal lengths for the WT and FWT. Three different L/H ratios were considered, L/H < 1, L/H = 1, and L/H > 1, as presented in Fig. 4.

### 2.2 Governing equations and solution method

The Reynolds-Averaged Navier–Stoke (RANS) equations are applied in the simulation. The conservation law of the energy, mass, and momentum of the working fluid was the source of these equations. Equations (1), (2) represent the conservative form continuity and momentum of the RANS equations for the two-dimensional steady incompressible flow [45,46]:

(1) $$\frac{\partial {\bar{u}}_{i}}{\partial x_{i}}=0$$

(2) $$\rho \frac{\partial {\bar{u}}_{i}{\bar{u}}_{j}}{\partial x_{j}}=-\frac{\partial \bar{p}}{\partial x_{i}}+\frac{\partial }{\partial x_{j}}\left(\mu \frac{\partial {\bar{u}}_{i}}{\partial x_{j}}-\rho \bar{{\overset{´}{u}}_{i}{\overset{´}{u}}_{j}}\right)+S_{i}$$

where the instantaneous value of velocity, *u* is expressed by the sum of its mean and the fluctuating part ${\bar{u}}_{i}$ and $\overset{´}{u_{i}}$ of *i* direction, respectively, as: $u={\bar{u}}_{i}$ + $\overset{´}{u_{i}}\text{,}$ $\bar{p}$ is the mean pressure, *μ* is the dynamic viscosity coefficient, *ρ* is the density, $\rho \bar{{\overset{´}{u}}_{i}{\overset{´}{u}}_{j}}$ is the Reynolds stress, and the generalized source term $S_{i}$ is the body force term. For the present case, the Mach number was less than 0.3, and the flow could be considered incompressible with constant viscosity; therefore, the energy equations were not solved.

The shear stress transport (SST) k-ω turbulence model comprises two equations formulated by Menter [47], which were revised by combining the standard k-ω model and k-ε models. Therefore, the SST k-ω model has greater accuracy in the near-wall region with the freestream independence of the k-ε model in the far-field regions. Better computational accuracy is achieved in capturing the physical flow and unfavorable pressure gradient by the suggested turbulence model SST k-ω, which also takes into account the impacts of turbulent shear stress transportation. The SST k-ω transport are expressed as equations (3) and (4) as shown below:

(3) $$\frac{\partial }{\partial x_{i}}\left(\rho ku_{i}\right)=\frac{\partial }{\partial x_{j}}\left(\Gamma_{k}\frac{\partial k}{{\partial x}_{j}}\right)+G_{k}-Y_{k}+S_{k}$$

and

(4) $$\frac{\partial }{\partial x_{j}}\left(\rho \omega u_{j}\right)=\frac{\partial }{\partial x_{j}}\left(\Gamma_{\omega }\frac{\partial \omega }{{\partial x}_{j}}\right)+G_{\omega }-Y_{\omega }+S_{\omega }$$

where:

*k* is the turbulence kinetic energy.

*ω* the specific dissipation rate.

*G*_*k*_ is the production of turbulence kinetic energy (KE) due to mean velocity gradients.

*G*_*ω*_ is the production of the dissipation rate.

*Γ*_*k*_ and *Γ*_*ω*_ are the effective diffusivity of k and ω, respectively.

*Y*_*k*_ and *Y*_*ω*_ are the dissipation of *k* and *ω*, respectively, due to turbulence.

*S*_*k*_ and *S*_*ω*_ are user-defined source terms.

Many researchers have attempted to evaluate the SST k-ω model's ability to accurately represent the attributes of dynamic stall. Daroczy et al. [48] compared five turbulent models commonly used in VAWT simulations using 2-D versus different experimental works to optimize the most suitable model. The results indicated that the k-ω SST model agreed with the experimental data. Rezaeiha et al. [49] analyzed seven commonly employed turbulence models in order to ascertain the model that exhibits the highest level of accuracy for a computational fluid dynamics (CFD) simulation of VAWTs. The investigation reached the conclusion that solely the SST models, which encompass the SST k-ω model, demonstrated satisfactory concurrence with the various experimental data examined and provided more precise predictions of the power coefficient throughout a broad range of TSR values. Thus, the turbulence model utilized in this investigation was the SST k-ω model. Moreover, the flow transition from the laminar to turbulent transitional SST k-ω model was employed in the current study to predict the flow during and after the stall angle at the last different AOAs to obtain accurate results. The present study employed ANSYS FLUENT, a commercial CFD tool, to solve the RANS equations. The solution techniques were configured according to the specifications specified in Table 1.

**Table 1:** Solution method.

Pressure-velocity coupling:	Coupled
Gradient:	Set as Least squares cell-based
Pressure, Momentum, Turbulent kinetic energy (k), and Specific dissipation rate (ω):	Set as Second-order upwind

### 2.3 Computational domain, meshing, and boundary conditions

For the static airfoil simulations conducted in this study, the physical properties of the fluid (air) are tabulated in Table 2. The Re_c_ = 180k, based on the airfoil chord length. The chord length (c) measured 0.14 m, indicating an incompressible flow as the Mach number was 0.06. Such conditions represent the values for small-to medium-scale urban VAWTs [7].

**Table 2:** Properties of the air.

Fluid Properties (air)	
T (C^o^)	20
U (m/s)	20
ρ (kg/m^3^)	1.204
Μ (kg.m/s)	1.855 x 10^−5^
Re	180k

**Fig. 5:**
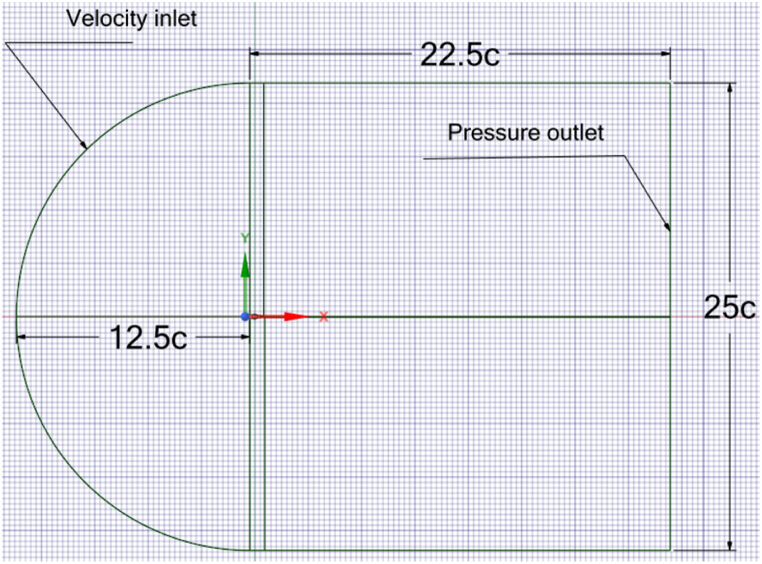
Computational domain dimensions and boundary conditions.

Various researchers have chosen a computational domain size ranging from 10 to 20 length/chord for their studies [35,[50], [51], [52]]. Their results revealed that this range is sufficient to obtain accurate aerodynamic results. In the present study, a bigger domain is adopted for greater accuracy and to capture the whole wake area behind the trailing edge within the domain. Fig. 5 presents the computational domain size adopted, and the boundary conditions set in the current study for the CFD simulation are shown in Table 3. The inlet boundary is at a distance of 12.5 times the chord length (12.5c) of the airfoil from the ¼ chord position. Similarly, the bottom and top boundaries are positioned 12.5c away from the trailing edge of the airfoil. The outlet boundary is situated at a distance of 22.5c from the ¼ chord position.

**Table 3:** Boundary conditions for CFD simulations.

Boundary	Type
Inlet (Top, Left, Bottom)	Velocity inlet, 20 m/s
Right	Pressure outlet, atmospheric pressure value
Airfoil and the WT surfaces	No-slip wall

**Fig. 6:**
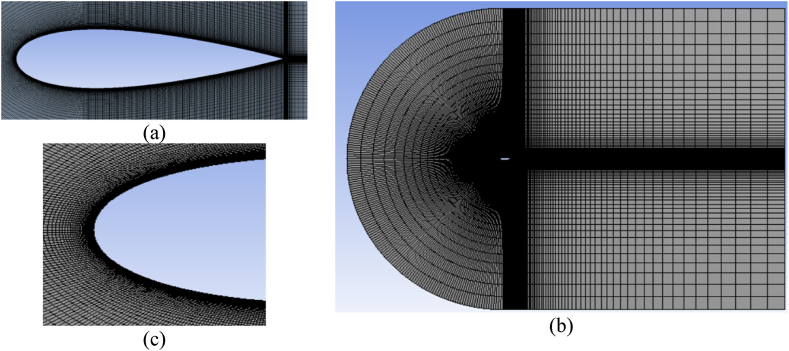
Details of the computational grid (a) Airfoil; (b) C-mesh computational domain (overall mesh); (c) Near the LE.

**Fig. 7:**
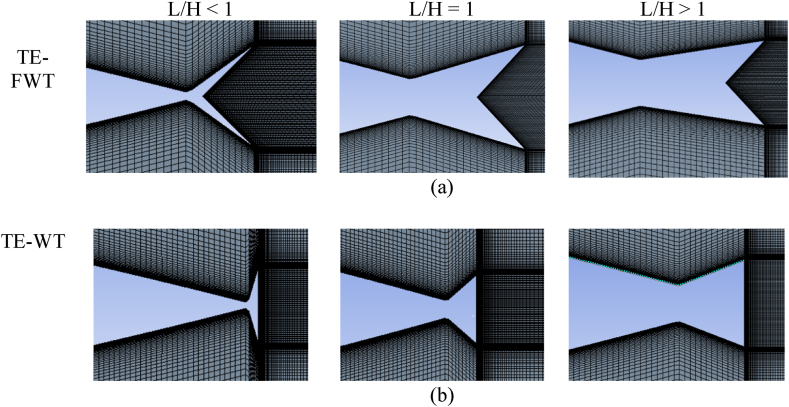
Details of the computational grid (a) Near the TE around FWT at L/H < 1, L/H = 1 and L/H > 1; (b) Near the TE around WT at L/H < 1, L/H = 1 and L/H > 1.

Various mesh attributes enhance mesh quality, resulting in better accuracy of calculation outcomes by minimizing computing time wastage. The study by Lu et al. [53] comprehensively examined various mesh parameters and their impact on the accuracy of solution predictions. The present investigation employed a structured C-type mesh. The grid distribution near the airfoil surface, where the boundary layer was present, was appropriately enhanced to capture the flow dynamics around the airfoil precisely. This was achieved by considering the layer-wise growth rate, which determines the mesh size of the boundary layer and the initial mesh height in close proximity to the wall. A structured mesh was also constructed in the far field within the wake zone. Fig. 6 depicts the airfoil (Fig. 6(a), the complete computational domain (Fig. 6(b)), and a magnified representation of the leading edge (Fig. 6(c). In the meantime, Fig. 7 depicts the computational grid in the vicinity of the TE-WT and TE-FWT, encompassing various ratios employed, namely the L/H = 1 instance, L/H < 1 (Fig. 7(a), and L/H > 1 (Fig. 7(b)).

### 2.4 Grid dependency test

**Fig. 8:**
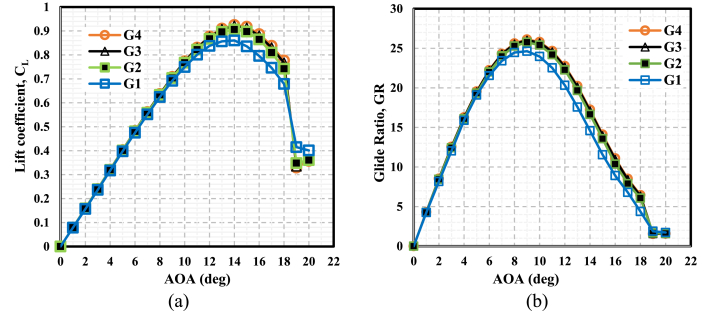
Comparison of CFD simulation for four grid distributions for the clean static airfoil. (a) Lift (b) Glide ratio.

This simulation examined four distinct grid configurations in order to assess the extent to which the choice of grid influenced the findings. Table 4 displays the specifics of the grids that were examined at a Reynolds number of 180,000. Fig. 8 depicts the fluctuations of the C_L_ (Fig. 8(a)) and the glide ratio (Fig. 8(b)) across a broad range of AOAs for the grid distributions denoted as G1, G2, G3, and G4. The lift coefficient exhibited an increase as the grid refinement progressed from G1 to G3. However, it was observed that G4 did not provide any further enhancements in lift despite utilizing a high-concentrating mesh. This suggests that the simulation outcomes remained unaffected by the grid for the chosen cell numbers. As a result, the clean airfoil configuration with approximately 108,000 cells, denoted as G3 was selected for implementation in the present simulation. In the context of the WTs, the airfoil utilized around 117,800 grid cells. As depicted in Fig. 6, the airfoil exhibits a distribution of 800 nodes along its chord line, with a greater level of mesh refinement observed in the vicinity of the LE and TE regions on both the upper and lower sides of the airfoil. The total number of layers in the system is 100, with the initial height of the first layer being 0.016 mm and a growth rate of 1.097. The non-dimensional distance, denoted as y^+^, of the initial grid points was maintained at around 1. This indicates that the mesh near the wall was adequately polished to improve its capacity to represent the viscous sublayer accurately.

**Table 4:** Grid parameters of the clean static NACA 0021 airfoil.

No	Grid Name	y+	Number of Cells
1	G1	≤1	27,300
2	G2	≤1	64,000
3	G3	≤1	108,000
4	G4	≤1	130,900

## 3 Results and discussion

### 3.1 CFD validation

**Fig. 9:**
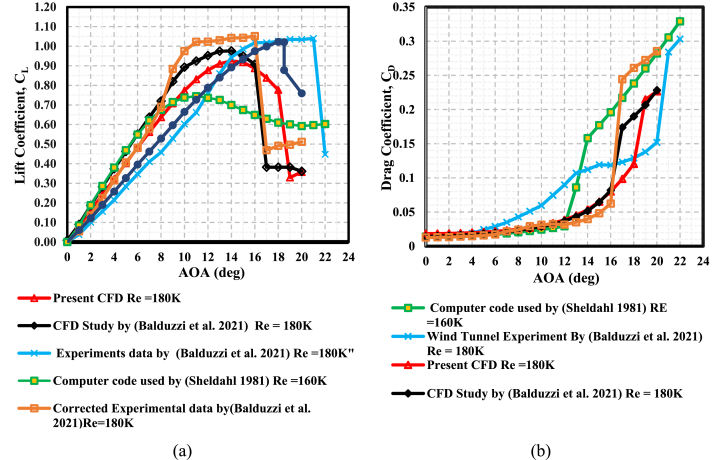
Validation of static polars (NACA 0021 profile) up to stall angle against literature data at Re∼180K **(a)** Lift coefficient (**b)** Drag coefficient.

The current simulations were validated by the comparison of the static airfoil aerodynamic characteristics (C_L_ and C_D_) from previous CFD simulations and measurements from wind tunnels that had used the same airfoil details (c = 0.14 m, Re_c_ = 180k, and NACA 0021) with the present study. Fig. 9(a) and (b) enable a comparison of C_L_ and C_D_ from the experimental and computational data for the NACA 0021. The referred data utilized for validation of this study were sourced from reputable publications, including the Sandia National Laboratories Energy report authored by Sheldahl and Klimas [54], the NACA technical report by Jacobs [55], and the recent work conducted by Balduzzi et al. [37], which involved both computational fluid dynamics (CFD) simulations and experimental investigations, incorporating certain modifications to the experimental data.

Despite variations of wind tunnel and varying experimental conditions, a majority of the studies documented in the literature have exhibited satisfactory alignment with the present study concerning the static stall angle and the curve's slope. However, it is worth noting that the data acquired by Sheldahl and Klimas, which were derived through the utilization of the PROFILE code, deviated from this trend. The stall angle in this work did not appear with a nearly constant lift at AOAs between 16^օ^ and 20^օ^. The angle of lift coefficient reduction in the current CFD simulation matched well with Jacobs's reduction in the lift coefficient. The drag coefficient values obtained from the present simulation exhibited satisfactory concurrence with the data reported in the existing literature up until the occurrence of static stall. Typically, airfoil stalling occurs when the AOA is around 15^օ^. Balduzzi's CFD work visualized a nearly constant lift after stalling at AOAs between 16^օ^ and 20^օ^, while the current study showed a constant lift at AOAs of 19^օ^ to 20^օ^. The occurrence can be attributed to a persistent trailing edge stall along with dramatic increases in drag due to separation in the boundary layer. The experimental work by Balduzzi and Jacobs showed AOA stalling beyond 20^օ^.

In the present study, the general behavior of the lift coefficient indicated good agreement with Balduzzi's work despite the slightly underestimated maximum lift coefficient by the CFD simulation. The observed phenomenon can potentially be ascribed to the diverse domain topology, the incomplete turbulence model, and the assumption of totally turbulent flow in the current CFD simulation.

### 3.2 Effects of the WT and FWT heights

The effects of the WT heights attached to the NACA 0021 airfoil (two-sided WFs with a mounting angle γ = 90^օ^) and FWT (two-sided WFs with an inclined angle of 45^օ^ were investigated. In this section, the length, L, and height, H of WF dimensions were considered to have the same length. The effects on the C_L_ and C_D_ are discussed in the following sections.

#### 3.2.1 Effects on the lift and drag coefficients

**Fig. 10:**
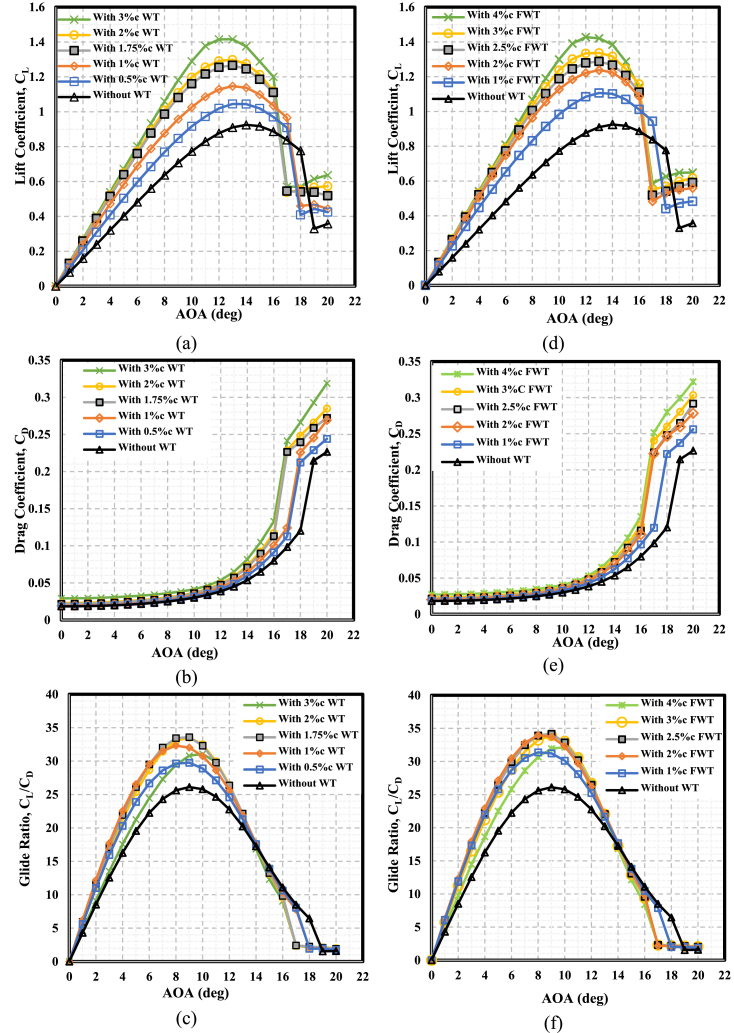
Effect of WT and FWT heights on the aerodynamics of the NACA 0021 airfoil. **At the lift side with** TE-WT **(a)** Lift **(b)** Drag **(c)** Glide ratio**. At the right side** with TE-FWT**(d)** Lift **(e)** Drag **(f)** Glide ratio.

This section describes the effects of the WT and FWT heights on the NACA 0021 airfoil. Fig. 10(a) and (d) present the increase in the lift coefficient with the WF height increases for the TE-WT and TE-FWT, respectively, at the AOAs from 0^օ^ to 20^օ^. The lift force increased linearly with AOAs of up to 12^օ^ and became steeper at greater WT heights, reaching the maximum values at AOAs of (12^օ^ to 14^օ^). After this point, the lift coefficient decreased with further AOA increases. Then, as the critical AOA exceeded, a sudden reduction in the lift occurred, which is known as a stall due to the boundary layer separation from the airfoil. After stalling, the lift appeared nearly constant, as shown in Fig. 10.

Both WTs shifted the C_L_− AOA curve to lift by 1^°^ compared to the clean airfoil. The increments in the maximum C_L_ values with the WT and FWT heights are presented in Table 5. The lift was enhanced as the WT height grew because the airfoil's effective camber also increased. This greater pressure differential between the upper and lower airfoil surfaces led to an increase in lift. The C_L_ increased proportionally with the WT height increments.

**Table 5:** Increments of the maximum value of lift coefficient for different TE-WT and TE-FWT heights in comparison with the clean airfoil case.

Airfoil with wedge tail (TE-WT)		Airfoil with fish wedge tail (TE-FWT)		AOA at Maximum C_L_ (°)	
WT height (H)	Increments in maximum C_L_ (%)	FWT height (H)	Increments in maximum C_L_ (%)	TE-WT	TE-FWT
Clean airfoil	_	Clean airfoil	_	14	14
0.5%c WT	12.93	1%c FWT	19.61	13	13
1%c WT	23.98	2%c FWT	33.80	13	13
1.75%c WT	36.92	2.5%c FWT	39.18	13	13
2%c WT	40.37	3%c FWT	44.51	13	13
3%c WT	53.05	4%c FWT	54.34	13	12

The value of the maximum C_L_ increased by 12.93 % at a WF height of 0.5%c to 53.05 % at a WF height of 3%c for the TE-WT case compared to the clean airfoil case. Meanwhile, in the case of the TE-FWT, the maximum value of C_L_ increased from 19.61 % at a WF height of 1%c to 54.34 % at a WF height of 4%c, compared to the clean airfoil. Effects on the drag coefficient.

The effects on the drag coefficient at a wide range of AOAs using various TE-WT and TE-FWT heights are shown in Fig. 10(b) and (e), respectively. At AOAs <10°, the drag slightly increased; meanwhile, at AOAs >10°, the increments were steeper due to the boundary layer separation from the airfoil at the stall angles. Furthermore, as the height of the WTs increased, the drag penalty saw significant proportionate increases.

#### 3.2.2 Glide ratio, C_L_/C_D_

Fig. 10(c) and (f) show the differences in glide ratio between the AOAs and the clean airfoil at various TE-WT and TE-FWT heights, respectively. The lift coefficient increased linearly for low to moderate AOAs, whereas the glide ratio increased dramatically with a little drag penalty. The glide ratio dropped while there was a significant drag penalty at medium to high AOAs.

**Fig. 11:**
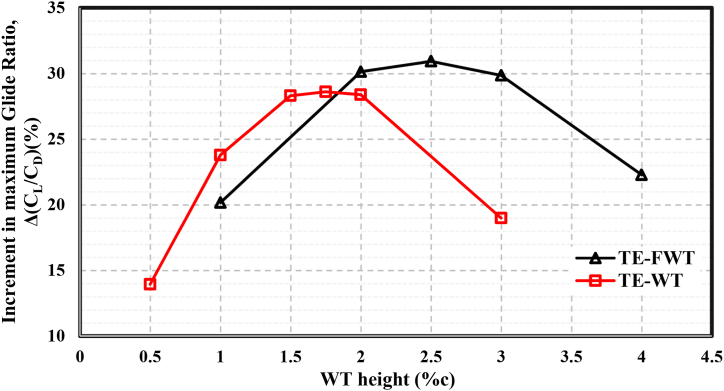
Percentage of increase in maximum glide ratios at different WT and FWT heights compared with the clean airfoil.
• **Case of TE-FWT**: The glide ratio values increased, as illustrated in Fig. 10(f), with FWT height increases of %1c, %2c, and %2.5c. Meanwhile, at FWT heights of 3%c and 4%c, the glide ratios showed reductions in the maximum values due to the large increase in drag. Thus, the optimal FWT height was H = 2.5%c with an increase in the maximum glide ratio value of 30.93 % at AOA = 9°, compared to the clean airfoil case, as shown in Fig. 11.

• **Case of TE-WT**: The maximum glide ratio increased, as illustrated in Fig. 10(c), with WT heights of %0.5c, %1c, and %1.75c. Meanwhile, the WT heights of 2%c and 3%c showed reductions in the maximum values due to the significant drag penalty corresponding to the WT height increases. This might have been due to the height of the boundary layer thickness being exceeded. Consequently, in comparison to the clean airfoil situation, the optimal WT height was H = 1.75%c (or 3.5%c for the entire WT height), with an increase in the maximum glide ratio value of 28.60 % at AOA = 9°, as illustrated in Fig. 11.

The NACA 0021 equipped with the TE-FWT performed better than the TE-WT. From Fig. 11, it can also be concluded that the point of intersection of the TE-WT and TE-FWT curves meant that the glide ratio increments of 28.57 %, in comparison with the clean airfoil case, could be achieved using either type of wedge tail with a height at about 1.83 % of the chord length.

### 3.3 Effects of the WT and FWT length

**Fig. 12:**
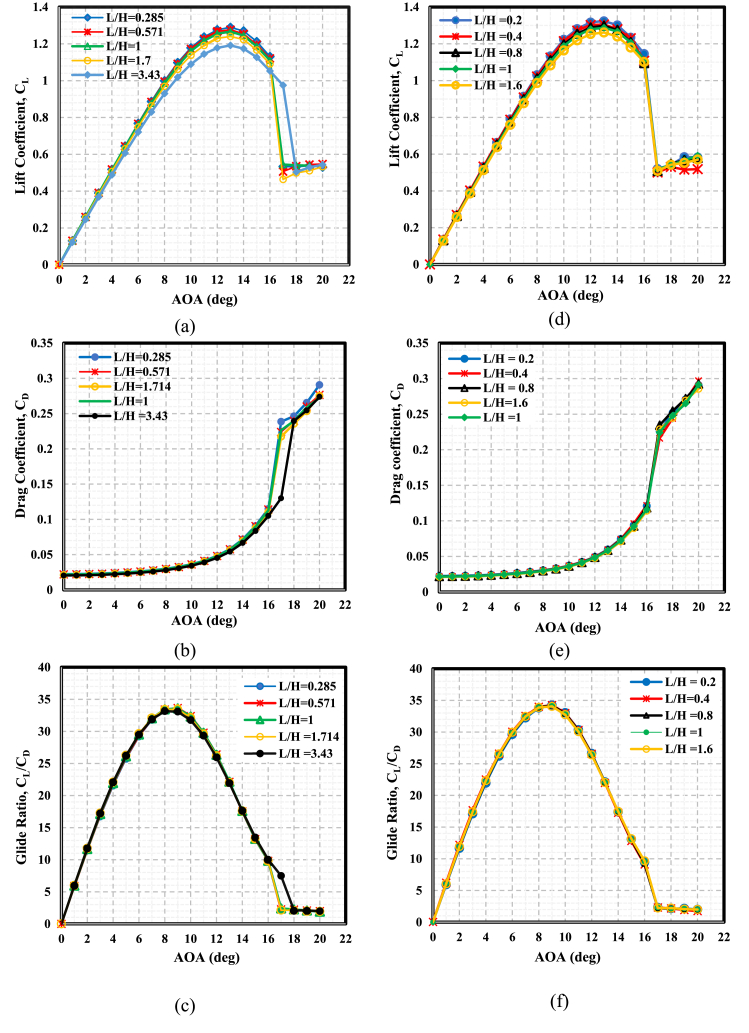
Effect of WT and FWT lengths on the aerodynamics of the NACA 0021 airfoil. **At the lift side with** TE-WT **(a)** Lift **(b)** Drag **(c)** Glide ratio**. At the right side** with TE-FWT **(d)** Lift **(e)** Drag **(f)** Glide ratio.

**Fig. 13:**
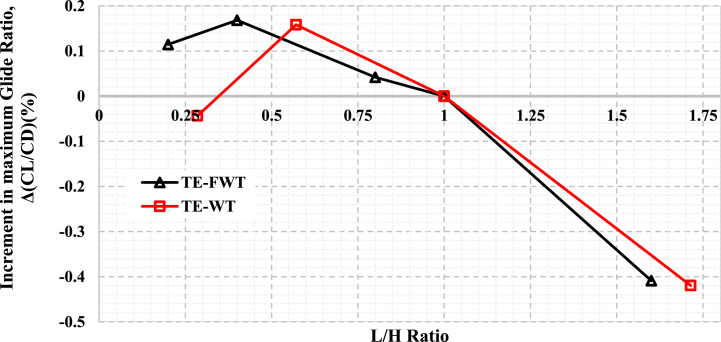
Percentage of increase in maximum glide ratio at different WT and FWT lengths compared with the L/H = 1 case.

This section outlines the effects of WT and FWT lengths at the optimal height on the aerodynamics of a static NACA 0021. The optimal height was determined from the previous section, i.e., L/H = 1). Fig. 12 and Table 6 show that several L/H ratios were investigated at L/H < 1 and L/H > 1 in comparison to the optimal WT and FWT heights of 1.75 and 2.5 %, respectively. The C_L_-AOA curves in Fig. 12(a) and (d) showed positive increments in the maximum lift coefficient at L/H < 1, but at L/H > 1, the maximum C_L_ decreased. This might be explained by the increased drag cost brought on by the airfoil's longer upstream length. Moreover, the lift coefficient curves show the highest lift values at L = 0.5%c for both WT and FWT; however, this increase in lift was associated with a significant drag increase, as illustrated in Fig. 12(b) and (d). As a result, low glide ratio values are produced, as shown in Fig. 12(c) and (f). This reduction at L = 0.5%c, also presented in Fig. 13, might be attributed to the vortex tip losses at the TE wedge tips, particularly for thin flaps L/H « 1. The increase of the L/H ratio at both the lower and higher surfaces of the airfoil's trailing edge resulted in a reduction in drag. Simultaneously, the glide ratio experienced a rise, ultimately reaching its peak increment values. Therefore, the maximum glide ratio increments achieved, compared to the L/H = 1 case, were 0.16 % for the TE-WT, which was reached at L/H = 0.285 (L = 1%c, H = 1.75%c), and 0.17 % for the TE-FWT, which was achieved at L/H = 0.4 (L = 1%c, H = 2.5%c).

**Table 6:** Increment in the maximum glide ratio at constant WT height and with different WT lengths for TE-WT at 1.75%c (2.45 mm) and TE-FWT at 2.5%c (3.5 mm) in comparison with the L/H = 1 case.

	TE-WT at H = 1.75%c				TE-FWT at H = 2.5%c				
WF based on chord length (%)	0.5%c	1%c	1.75%c	3%c	0.5%c	1%c	2%c	2.5%c	4%c
WF length L(mm)	0.7	1.4	2.45	4.2	0.7	2.8	3.5	4.2	5.6
L/H ratio	0.285	0.571	1	1.714	0.2	0.4	0.8	1	1.6
Increments in maximum C_L_ (%)	1.84	0.85	0	−2.06	2.83	1.35	0.84	0	0
Increment in maximum Glide Ratio, C_L_/C_D_ (%)	−0.044	0.16	0	−0.42	0.115	0.17	0.042	−2.01	−0.409

In general, it was observed that altering the wedge length when L/H < 1 yielded marginal enhancements in aerodynamic performance relative to the scenario where L/H = 1. In contrast, at L/H > 1, the cases showed rapid falls in glide ratios. From this study, the furthest L/H < 1 for TE-WT is 0.571, and TE-FWT is 0.4, both at the length of 1%c at the respective optimal height. Further reduction of L/H beyond these points is not preferable as the C_L_ increments show a declination trend.

### 3.4 Aerodynamic characteristics for the optimal TE-WT and TE-FWT

**Fig. 14:**
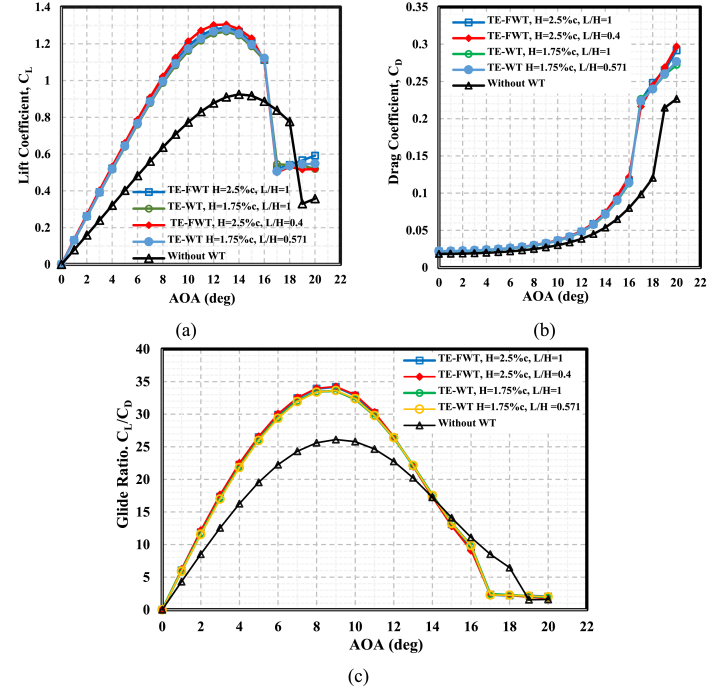
Optimal aerodynamic characteristics for TE-WT and TE-FWT cases compared to the clean NACA 0021 case (**a)** Lift **(b)** Drag **(c)** Aerodynamic efficiency.

This section presents the maximum aerodynamic characteristics for the four cases resulting from the previous sections, including the effects of height and length: the TE-WT with a height of 1.75%c at L = H and L = 1%c and the TE-FWT with a height of 2.5%c at L = H and L = 1%c. These were compared to the clean airfoil case, as shown in Fig. 14. In Fig. 14(a), the TE-FWT at L/H < 1 showed the maximum C_L_ at the optimal height (2.5%c) and length (1%c), respectively, with the highest C_L_ of 1.30 at AOA = 13°. Overall, the maximum lift increased by approximately 41 % compared to the clean airfoil case. The case of the TE-FWT (H = 2.5, L = 1%c, L/H = 0.4) showed the lowest drag coefficient at AOA <10° before it increased at higher AOAs, as shown in Fig. 14(b) and achieved the highest increments in glide ratio values, with 31.15 % at AOA = 9°, in comparison with the clean airfoil case, as presented in Fig. 14(c).

### 3.5 Pressure distribution

**Fig. 15:**
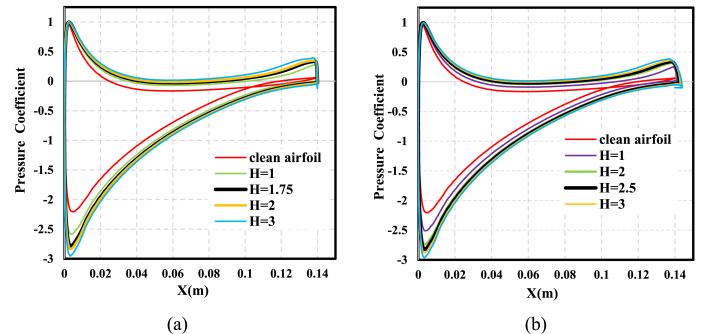
Pressure coefficient distribution for different TE-WT and TE-FWT heights compared to clean airfoil at AOA = 9°. (a) Pressure coefficient distribution for TE-WT; (b) pressure coefficient distribution for TE-FWT.

The variations in the pressure coefficient around the NACA 0021 airfoil surface along the chord length at AOA = 9° for different TE-WT and TE_FWT heights were obtained computationally for both airfoils, with and without wedge tails, as shown in Fig. 15. Increased suction on the upper airfoil surface and increased pressure on the lower airfoil surface obviously appeared with the addition of the WTs, which resulted in a lift improvement. The suction reached its maximum value close to the TE where the WT was mounted. As the WT height increased, so did the pressure differential between the airfoil's upper and lower surfaces. The pressure achieved its maximum near the LE of the airfoil. However, the rate of increment in the static pressure coefficient reduced as the WT height increased. Various TE configurations, including WT and FWT, are presented in Fig. 15(a) and (b), respectively, showing the clear effect on the pressure coefficient distribution at the TE. Because the FWT installed at an angle of 45° somewhat lengthened the airfoil beyond the TE and the WT at 90°, the pressure traveled downstream for the TE-FWT.

**Fig. 16:**
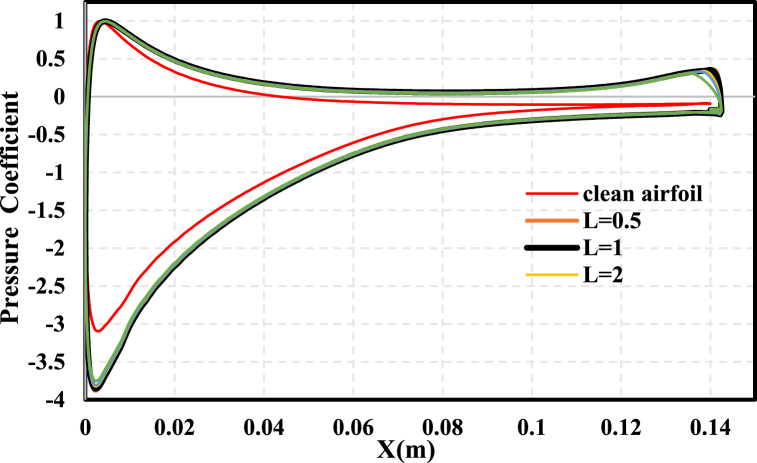
Pressure coefficient distribution for H = 2.5%c and different TE-FWT lengths compared to clean airfoil at AoA = 13^°^

Fig. 16 presents the pressure coefficient on the NACA 0021 airfoil surface with a constant TE-FWT height of 2.5%c and for different FWT lengths at AOA = 13. As the length extended upstream L > 1%c from the TE, the increments in suction and pressure fell at a very slow rate, up to the length where L/H = 1 at H = 2.5%c and decreased at a higher rate beyond L/H = 1.

### 3.6 Fish wedge tail versus fish Gurney tail

**Fig. 17:**
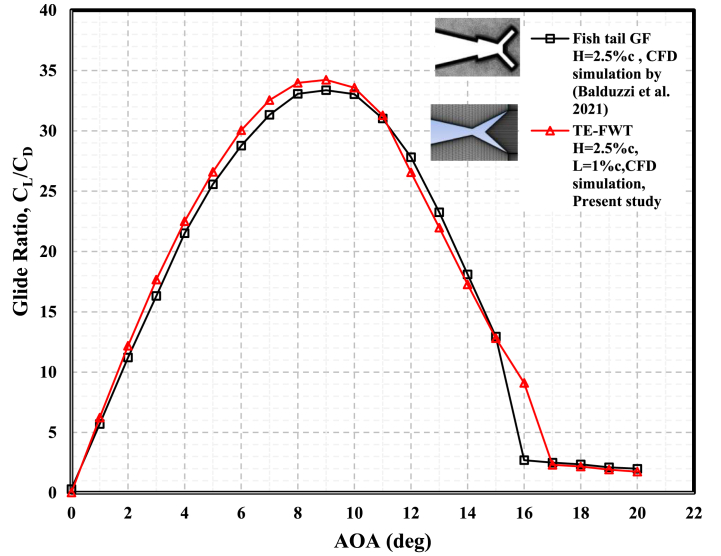
Comparing the maximum glide ratio for TE-FWT with H = 2.5%c, L = 1%c, and FGT with H = 2.5%c from the literature.

The flap wedge's dimensions (length and height) are the main features that determine its proper size and aerodynamic performance. At the same time, the GF effectiveness depends only on the height, so the Gurney flap represents a particular case of the TE wedge with L/c ≈ 0. Fig. 17 presents a comparison of the maximum glide ratios at a wide range of AOAs that were achieved in this study using an NACA 0021 equipped with a TE-FWT with an optimal height of 2.5%c and a length of 1%c versus the same airfoil section, chord length, and inclination angle of 45^օ^ equipped with a GF with a height of 2.5%c, as used in the study by Balduzzi et al. (2021). The aerodynamic performance of the TE-FWT was greater than that of the GF at AOAs <11°, while at AOAs >11°, the GF performed better before stalling at one degree of AOA earlier than the wedge tail. This performance improvement of the FWT over the GF was due to the length of the wedge, which prevented greater circulation in the case of the GF. Additionally, a wedge flap with L > 0.5%c could prevent tip loss vortices, as well as provide the blade with greater stability and mechanical stiffness.

### 3.7 Trailing-edge flow structure

**Fig. 18:**
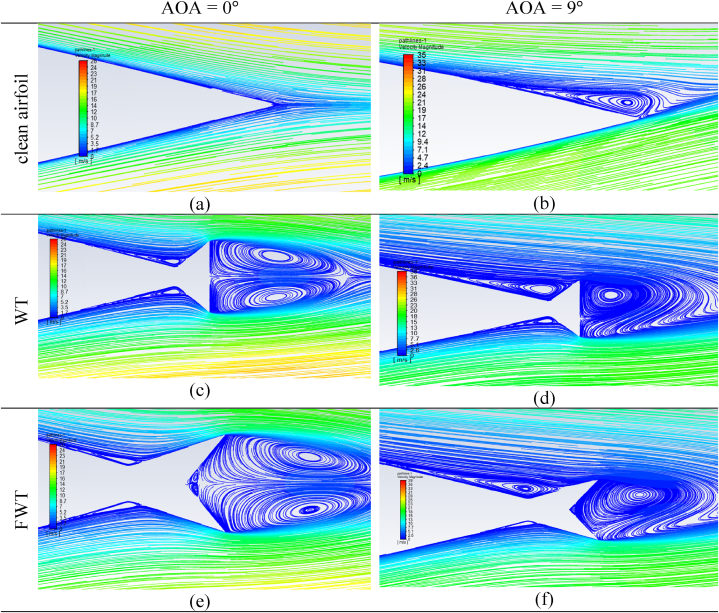
Streamlines for TE-WT and TE-FWT at different AOAs compared to a clean NACA 0021 airfoil. (a) Clean airfoil at AOA = 0°; (b) clean airfoil at AOA = 9°; (c) airfoil with WT at AOA = 0°; (d) airfoil with WT at AOA = 9°; (e) airfoil with FWT at AOA = 0°; (f) airfoil with FWT at AOA = 9°.

**Fig. 19:**
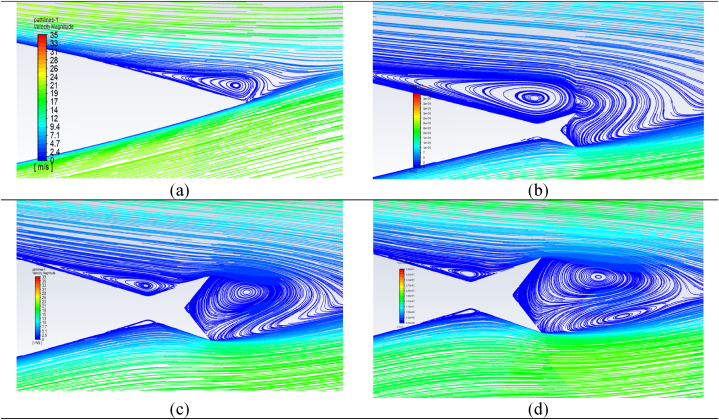
Streamlines for TE-FWT at different heights compared to a clean NACA 0021 airfoil at AOA = 9°. (a) Clean airfoil; (b) H = 1 %; (c) H = 2.5%c; (d) H = 4%c.

The flow structure near the TE of the NACA 0021 airfoil equipped with a WT and an FWT at AOAs of 0° and 9° was compared to the clean airfoil, as presented in Fig. 18. Two large counter-rotating vortices appearing at AOA = 0° in the wake of the TE-WT and the TE-FWT, as well as two other small counter-rotating vortices, are clearly visible downstream of the only TE-FWT due to their geometry. In addition to these vortices, two more vortice regions were created upstream of both WTs. At AOA = 9°, one vortex appeared near the TE in a clean airfoil, and two large counter-rotating vortices downstream for the WT and FWT merged into one vortex. The vortex on the upper surface upstream extended while the one on the lower surface reduced. Fig. 19 shows the flow structure near the TE of the airfoil equipped with the FWT at different heights in the case of L/H = 1. As the height of the wedge flap increased, the vortex strength increased, which led to more deflection of the flow at the TE towards the middle of the tails, thus increasing the effective downwash. Therefore, the vortices were mainly responsible for the pressure difference between the suction and pressure surfaces, hence the lift enhancements.

**Fig. 20:**
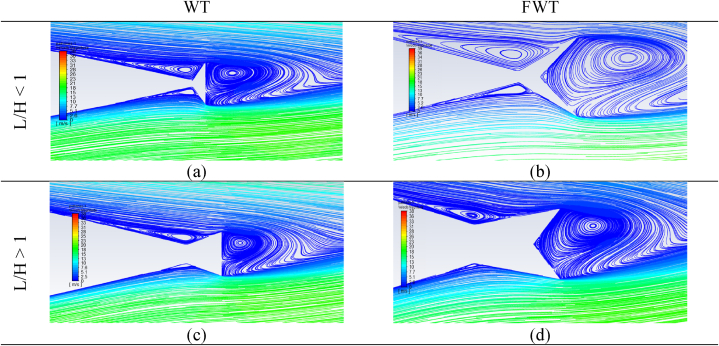
Streamlines for different tails at AOA = 9° and different L/H ratios compared to a clean NACA 0021 airfoil. (a) WT at L/H < 1; (b) FWT at L/H < 1; (c) WT at L/H > 1; (d) FWT at L/H > 1.

Fig. 20 shows the flow structure near the TE of the NACA 0021 airfoil equipped with a WT and an FWT at optimal heights and different lengths for the cases of L/H < 1 and L/H > 1. Both the WT and FWT showed higher vortice circulation at the upstream region in the case of L/H < 1. However, as the length of the wedges increased in the L/H > 1 case, the vortices in the upstream region diminished, especially the lower upstream vortex, resulting in a decrease in suction and, hence, a reduction in the lift enhancement.

## 4 Conclusion

This study's main objective was to evaluate the impact of the 2D NACA 0021 static airfoil, which is used with a TE-WT and a TE-FWT, on aerodynamic performance. Different cases were considered to investigate the influence of the TE-WT and TE-FWT heights, as well as the effect of the L/H ratios at the optimal height on the aerodynamic characteristics. The pressure distribution and the flow structure near the airfoil TE were also studied. The main findings are summarized as follows.

• The utilization of WTs on the TE of an airfoil resulted in enhanced aerodynamic performance across all examined scenarios, in contrast to the clean airfoil.
• The optimal WT and FWT heights at L/H = 1 were 1.75%c and 2.5%c, respectively.
• The optimal length of the WT and FWT at their optimal heights was 1%c to achieve the optimal dimensions in the L/H < 1 case.
• The total maximum increments in the glide ratio values at the optimal height and length of the TE-WT and TE-FWT were 28.80 % and 31.15 %, respectively, at AOA = 9^օ^, in comparison with the glide ratio of the clean airfoil.
• The WT height substantially impacts the aerodynamic performance, while its length has a minor influence.
• The NACA 0021 equipped with the FWT demonstrated aerodynamic performance that exceeded that of the WT by 8 %.
• Thin WTs with L < 1%c showed a slight degradation of the maximum glide ratio. This may be attributed to the vortex tip losses at the blade tips.
• The WTs with L/H < 1 showed a slight improvement in aerodynamic performance, while those at L/H > 1 showed a rapid decrease in performance compared with the L/H = 1 case.
• The trailing edge FWT performed better than the fish Gurney tail for the same airfoil section and Re number at AOA <11°.

The findings of the current study enable a better understanding of how equipping airfoils with WTs affects aerodynamic performance. However, the investigations were limited to Re = 180k, the NACA 0021 airfoil section, and the SST turbulence model. Further studies could be done to explore the effects of different Reynolds numbers, airfoil sections, and turbulence models.

Further research will be conducted to assess the impact of WTs and FWTs with optimal dimensions on the performance of VAWTs. This research aims to validate the extent of the enhancements achieved. WTs and FWTs with varying geometric characteristics will be installed on the blades. Moreover, WTs are commonly used in marine rudder applications, and the current study shows that the FWT increments in glide ratio exceeded the WT increments by over 8 % under aerodynamic forces. Further developments could include an FWT under hydrodynamic loads.

## Data availability statement

Data will be made available on request.

## Additional information

No additional information is available for this paper.

## CRediT authorship contribution statement

**Asmail A.M. Abdalkarem:** Data curation, Formal analysis, Investigation, Writing – original draft. **Roaa Ansaf:** Data curation, Methodology. **Wan Khairul Muzammil:** Conceptualization, Validation, Visualization, Writing – review & editing, Supervision. **Adnan Ibrahim:** Conceptualization, Resources. **Zambri Harun:** Resources, Software, Validation. **Ahmad Fazlizan:** Conceptualization, Funding acquisition, Project administration, Resources, Software, Supervision, Writing – review & editing.

## Declaration of competing interest

The authors declare the following financial interests/personal relationships which may be considered as potential competing interests:Ahmad Fazlizan reports financial support was provided by 10.13039/501100004515Universiti Kebangsaan Malaysia.

## Abbreviations

LE: Leading edge
TE: Trailing edge
GF: Gurney flap
WT: Wedge tail
FWT: Fish wedge tail
WF: Wedge flap
SST: Shear stress transport
AOA: Angle of attack
G: Grid distribution
TSR: Tip speed ratio
HAWT: Horizontal axis wind turbine
VAWT: Vertical axis wind turbine
NACA: National Advisory Committee for Aeronautics
RANS: Reynolds averaged Navier-stokes

**Symbols**
c: Chord length
Re: Reynolds number
L: Length
H: Height
C_L_: Lift coefficient
C_D_: Drag coefficient
y+: Non-dimensional wall distance
U: Free stream velocity
C_L_/C_D_: Glide ratio
γ: Wedge angle to the chord line
